# Immunosuppressed Miniswine as a Model for Testing Cell Therapy Success: Experience With Implants of Human Salivary Stem/Progenitor Cell Constructs

**DOI:** 10.3389/fmolb.2021.711602

**Published:** 2021-09-30

**Authors:** Danielle Wu, Isabelle M. A. Lombaert, Maximilien DeLeon, Swati Pradhan-Bhatt, Robert L Witt, Daniel Anton Harrington, Mark G Trombetta, Michael J Passineau, Mary C. Farach-Carson

**Affiliations:** ^1^ Department of Diagnostic and Biomedical Sciences, School of Dentistry, University of Texas Health Science Center at Houston, Houston, TX, United States; ^2^ Department of Bioengineering, Rice University, Houston, TX, United States; ^3^ Department of Biologic and Materials Sciences and Prosthodontics, School of Dentistry, University of Michigan, Ann Arbor, MI, United States; ^4^ Biointerfaces Institute, University of Michigan, Ann Arbor, MI, United States; ^5^ Helen F. Graham Cancer Center, Christiana Care Health System, Newark, DE, United States; ^6^ Department of Biosciences, Rice University, Houston, TX, United States; ^7^ Division of Radiation Oncology, Allegheny Health Network, Pittsburgh, PA, United States; ^8^ Gene Therapy Program, Allegheny Health Network, Pittsburgh, PA, United States

**Keywords:** miniswine, tissue engineering, immunosuppression, salivary gland, human stem cells

## Abstract

An urgent need exists to develop large animal models for preclinical testing of new cell therapies designed to replace lost or damaged tissues. Patients receiving irradiation for treatment of head and neck cancers frequently develop xerostomia/dry mouth, a condition that could one day be treated by cell therapy to repopulate functional saliva-producing cells. Using immunosuppression protocols developed for patients receiving whole face transplants, we successfully used immunosuppressed miniswine as a suitable host animal to evaluate the long-term stability, biocompatibility, and fate of matrix-modified hyaluronate (HA) hydrogel/bioscaffold materials containing encapsulated salivary human stem/progenitor cells (hS/PCs). An initial biocompatibility test was conducted in parotids of untreated miniswine. Subsequent experiments using hS/PC-laden hydrogels were performed in animals, beginning an immunosuppression regimen on the day of surgery. Implant sites included the kidney capsule for viability testing and the parotid gland for biointegration time periods up to eight weeks. No transplant rejection was seen in any animal assessed by analysis of the tissues near the site of the implants. First-generation implants containing only cells in hydrogel proved difficult to handle in the surgical suite and were modified to adhere to a porcine small intestinal submucosa (SIS) membrane for improved handling and could be delivered through the da Vinci surgical system. Several different surgical techniques were assessed using the second-generation 3D-salivary tissue (3D-ST) for ease and stability both on the kidney capsule and in the capsule-less parotid gland. For the kidney, sliding the implant under the capsule membrane and quick stitching proved superior to other methods. For the parotid gland, creation of a tissue “pocket” for placement and immediate multilayer tissue closure were well tolerated with minimal tissue damage. Surgical clips were placed as fiduciary markers for tissue harvest. Some implant experiments were conducted with miniswine 90 days post-irradiation when salivation decreased significantly. Sufficient parotid tissue remained to allow implant placement, and animals tolerated immunosuppression. In all experiments, viability of implanted hS/PCs was high with clear signs of both vascular and nervous system integration in the parotid implants. We thus conclude that the immunosuppressed miniswine is a high-value emerging model for testing human implants prior to first-in-human trials.

## Introduction

Xerostomia, the subjective perception of dry mouth due to hyposalivation, is a serious oral morbidity affecting millions of people worldwide. Irradiation-induced xerostomia has received considerable attention because of its prevalence after radiotherapy for locally invasive cancers of the head and neck (Vissink et al., 2010). The precise prevalence of this condition is unknown, but extrapolation of public health figures for upper-airway cancers indicates that over 50,000 new cases/year occur in the U.S. alone. Globally, new cases sum up to 650,000 with 330,000 deaths (Bray et al., 2018). Unfortunately, xerostomia persists despite the introduction of intensity-modulated radiation therapy (IMRT) as a means of sparing the salivary glands from irradiation damage. At any one time, it is estimated that more than 150,000 Americans suffer from some degree of irradiation-induced xerostomia, and millions of other individuals suffer from xerostomia attributed to other causes including age, injury, and autoimmune disease (Maeshima et al., 2013). The latter group will benefit from the ability to allograft replacement tissues.

Radiation in the format of photons, electrons, or neutrons leads to irreversible xerostomia because of the widespread death of saliva-producing serous acinar cells. While the mechanisms leading to cell death remain debated and an active area of study reviewed in the work of Grundmann et al. (2009), it is widely agreed that in humans, chronic loss of salivary function post-radiation is a major problem for many cancer survivors. Measurable structural changes in tissue post-irradiation include periductal fibrosis, fat replacement, lymphocytosis, and destruction of the surrounding capillary vascular structure (Fajardo and Berthrong, 1981; Pandya et al., 2014). In addition to loss of water secretion into the oral cavity, irradiation-induced acinar cell loss impairs functions that greatly reduce patient quality of life posttreatment: 1) decreased production of salivary proteins including amylase and immunoglobulin A, 2) increased concentration of electrolytes, 3) increased salivary viscosity, and 4) decreased pH (Dreizen et al., 1976; Marks et al., 1981). Ineffective palliative therapies include water drinking, oral sialagogues, oral wash preparations, and salivary substitutes such as carboxymethylcellulose. Similarly, the cholinergic agonist pilocarpine has shown limited success in the treatment of radiation-induced xerostomia (Davies and Thompson, 2015).

To take a step toward a permanent cure, we developed a new large animal model for testing cell therapy for dry mouth disorders—the irradiated, immunosuppressed miniswine. This model can provide a new human-in-miniswine prototype to accurately test the ability of engineered salivary gland cell-based implants to provide permanent relief for human patients suffering from xerostomia subsequent to irradiation of the head and neck region. If successful, it is highly likely that this emerging testing platform can achieve broad utility as a large animal model that is suitable for regulatory agencies and for investigating other new therapies and/or devices built to contain human stem/progenitor cells (hS/PCs) from other tissues/organs. In work reported here for the first time, we show that the parent hyaluronate (HA)-based materials that we chose to encapsulate hS/PCs are biocompatible in immunocompetent porcine model hosts. We also show that immunosuppressed miniswine display no tissue reaction to encapsulated hS/PCs in migration-permissive hydrogels over a multi-week implant period during which the implanted cells remain viable. Because the irradiated miniswine salivary bed accurately reproduces the morphology and regenerative conditions in the human bed post-irradiation, it is a suitable and paradigm-changing model that can be used to test various implant prototypes using salivary-derived adult hS/PC populations to reestablish salivary functions.

## Materials and Equipment

### Animals

All procedures were conducted to ensure the most humane use of animals possible with care exercised at every step to ensure minimum pain and distress. All procedures were approved by the IACUC at the Allegheny Health Network (AHN) and followed or superseded guidelines established for use of large animal models in research. Male and female miniswine of the Yucatan strain, aged 3–4 months (20–35 lbs) at the start of the experiments, were used in these studies. All animals were purchased from Sinclair Bio-resources (Auxvasse, MO) and acclimated for at least 2 weeks after delivery before being irradiated or subjected to any surgical treatments. All caging was consistent with the standards specified in the Guide for the Care and Use of Laboratory Animals and the Animal Welfare Act. Post-surgery, animals were housed singly for 2 weeks until wounds were completely closed.

### 3D-Salivary Tissues

The 3D-ST implant consisted of a migration-permissive hyaluronate (HA) hydrogel affixed to a shaped commercial DynaMatrix^®^ Small Intestinal Submucosa (SIS) membrane (10.405.3040, Keystone Dental, Burlington, MA). For various types of *in vivo* testing, both acellular and cell-laden versions were created. An early 3D-ST prototype testing DynaMatrix^®^ and hydrogel interactions in culture over time used encapsulated fluorescent microspheres (10 µm, F8836 and 200 nm, F8763 Fluospheres^®^ Invitrogen) to delineate the hydrogel fraction of the 3D-ST from the SIS membrane. The basic hydrogel used for biomaterials testing was a thiolated HA (HA-SH) crosslinked with poly(ethylene glycol) diacrylate (PEGDA), that is, HA-SH/PEGDA gels (Glycosil, ESI BIO). For cell encapsulation studies, peptides (GRGDS and GGGPQ↓IWGQGK, GenScript) were conjugated to poly(ethylene glycol) (Ac-PEG-SVA, 3.4kDa, LysanBio). Migration permissiveness in hydrogel was provided by incorporating pendant acrylated-PEG-GRGDS and Ac-PEG-PQ-PEG-Ac with thiol-modified hyaluronic acid (Glycosil, ESI BIO) with varying thiol–acrylate molar ratios (SH:Ac, 6:1, 3:1) to control crosslinking densities (Hubka et al., 2019). For cell-based implant studies, hydrogels were gelled directly atop DynaMatrix^®^.

### Human Salivary Stem/Progenitor Cells

Human salivary tissues (parotid and submandibular) are collected from consented head and neck cancer patients (males and females) undergoing surgery at one of our collection sites. Freshly resected tissue is processed and explanted and hS/PCs are expanded and isolated as described previously in detail (Wu et al., 2018). All procedures were performed following the approved guidelines of the Institutional Review Boards at the cooperating collection sites. Lentiviral particles to transduce hS/PCs for stable expression of Gaussia Luciferase (GLuc, LTGR002, G & P Biosciences) were used prior to implantation in most parotid sites.

### Reagents (Immunosuppression/Tissue Processing/Analysis)

The reagents used for immunosuppression are listed in Table 1, along with the chosen regimen. Reagents for tissue processing and analysis included Live/Dead solution (Calcein AM, 80011, Biotium; Ethidium Homodimer III, 40050, Biotium; Hoechst 33342, ENZ-52401, Enzo) for immediate assessment of fresh tissue samples and optimal cutting temperature O.C.T compound (4583, Tissue-Tek) for tissue embedding and cryopreservation. Immunohistochemistry used the following antibodies: human nuclear antigen (MAB1281, Sigma), α-amylase (A8273, Sigma), CD31 (MA513188, Invitrogen), and beta III tubulin (ab18207, Abcam). Human α-amylase (AMY1 ELISA kit, EKC32542, Biomatik) and Gaussia Luciferase (ab189814, Abcam) were measured in collected samples as needed.

**TABLE 1:** Immunosuppression and health regimen for hS/PC-implanted miniswine.

Drug/supplement	Dose/route	Duration	Purpose
Tacrolimus	5 mg PO^a^; BID^b^	Morning of surgery; duration of protocol	Immunosuppression
Meloxicam	7.5 mg PO/once daily	Morning of surgery, continuing for 4 days post operation	Anti-inflammatory
Buprenorphine	0.01 mg/kg IM^c^	Once at induction	Pain relief
Iron	100 mg IM	Once during recovery	Nutritional supplement
Selenium and vitamin E^d^	1 ml/40 lb body weight IM	Once during recovery	Nutritional supplement
Mycophenolate mofetil	500 mg PO BID	Morning of surgery; duration of protocol	Antifungal
SMZ-TMP	30–45 mg/kg PO/once daily	Day after surgery; duration of protocol	Antibacterial
Ceftriaxone	25 mg/kg IV^e^ during surgery, given over 30 min	One time	Antibacterial

a PO (*per os;* by mouth).

b BID (*bis in die*; twice a day).

c IM (intramuscularly).

d Product name Bo-Se (Merck).

e IV (intravenously).

### Equipment

The AHN Large Animal Research Facility is AAALAC accredited and USDA inspected and occupies 3,230 ft^2^ of laboratory and operating room space. The facility holds three theater-sized operating rooms, a cardiac catheterization laboratory, and an intensive care unit. The facility is connected, *via* a dedicated elevator, to a 9,769-square-foot housing facility for large research animals. Operating rooms are equipped with inhalation anesthetic equipment, fluid management delivery systems, patient-warming devices, pressure- and EKG-monitoring capabilities, pulse oximetry, defibrillators, electrocautery, a clinical laboratory, steam sterilization, decontamination areas, and a surgical scrub area. A dedicated and highly experienced team of surgical technicians provides support for the surgical procedures. The recovery facility is equipped with two intensive care cages capable of maintaining a sterile atmosphere with temperature and oxygen control. While in the ICU, animals can be monitored with EKG and pulse oxygenation and can be maintained on IV fluids. The facility also contains a blood gas monitor, glucose monitoring equipment, and a cardiac defibrillator. For studies involving implant on the kidney capsule, a da Vinci Xi surgical system (Intuitive Surgical, Sunnyvale, CA) was used. For studies involving irradiated glands, a Siemens Primus^®^ (Concord, CA) linear accelerator was used. A Cerrobend^®^ (Bolton Metal Products, Bellefonte, PA) allow was used to block the electron beam from reaching structures outside the desired field of irradiation.

## Methods

### Materials Testing

Initial testing of biomaterial compatibility was conducted using immune competent miniswine. HA-SH/PEGDA hydrogels, approximately 1 ml of gel/implant, were implanted in surgical pockets placed in the minipig salivary bed. After 1 week, implants were removed, fixed in paraformaldehyde, paraffin embedded, sectioned, and analyzed for tissue reaction by a licensed pathologist at Christiana Health Care System (CCHS) (Newark, DE).

### hS/PC Encapsulation and 3D-ST Preparation

This work involved two collaborating sites separated by approximately 1,300 miles; thus, methods to encapsulate hS/PCs in stable hydrogels that could be shipped overnight from Houston, TX, to Pittsburgh, PA, for implant into miniswine were developed. 3D-STs were prepared in Houston by encapsulating well-described hS/PCs (Srinivasan et al., 2017) in migration-permissive hydrogels 7–10 days before shipment. Cell number was adjusted according to the volume of the hydrogel and typically ranged from 2 to 5M cells/ml of hydrogel. In previous work, we reported on the growth and dynamic behavior of these cells in hydrogels where they assemble, rotate, and even merge after encapsulation (Briese et al., 1991; Pradhan-Bhatt et al., 2013). After initial experiments with unsupported hydrogels, all subsequent experiments used hydrogels that were 2 cm in diameter and 5 mm in thickness that were gelled directly on cut, circular DynaMatrix^®^ SIS membranes in 12-well plates. 3D-STs were cultured as described (Wu et al., 2018) in 12-well plates, over which time the gels remained intact. By the time of shipment, hS/PCs had assembled into spherical structures averaging 30–50 µm in diameter. 3D-STs in plates filled with medium were placed in sealed containers and priority-shipped overnight by commercial carrier to Pittsburgh where upon arrival, they were transferred into cell culture plates with fresh medium and into proper culture conditions. For experiments in the parotid, the night before implant into miniswine, some hS/PCs were infected with lentivirus expressing GLuc as a means to readily follow secretion of hS/PC products into collected saliva post-implant. Reporter expression was confirmed in cell-conditioned medium the next morning prior to implant using a Turner Biosystems 20/20 Luminometer. Biosafety approvals are in place for use of these constructs at both performance sites.

Prior to implant, all 3D-STs were washed with sterile 1XPBS and fresh media lacking phenol red and transported sterilely in 12-well plates to the operating theater where they could be passed to and implanted by the surgeons.

### Irradiation Protocols for Induction of Hyposalivation

Miniswine were anesthetized with isoflurane using an induction chamber into which they enter voluntarily, a process that greatly reduces distress before ketamine/xylazine injections. After anesthesia and prior to irradiation, miniswine subjects received an intramuscular (IM) injection of ketamine (20 mg/kg) and xylazine (2 mg/kg) and were then placed in half-body vac-bags, and echosialography was performed to confirm the location of the parotid gland and to tattoo the overlying skin for beam targeting. The irradiation plan was built on protocols described earlier (Wang et al., 2015; Lombaert et al., 2020). Animals were anesthetized and irradiated once using a 12-MeV electron beam restricted to the right salivary gland, delivering a single fraction of 15 Gy following CT-based planning. This irradiation protocol was well tolerated with minimal side effects and little irradiation burn. Noninvasive *in vivo* imaging was performed post-irradiation to show that this dosing protocol delivered only minor macroscopic damage to the targeted gland, sparing the other side (not shown).

### Surgical Methods

#### Kidney Capsule Implantation

The kidney capsule is a well-accepted site for testing grafted tissue compatibility and viability (Cunha and Baskin, 2016) and was used here for initial viability studies in our immunosuppression model and to ensure that the animals would tolerate the hS/PCs under immunosuppression. Four-arm robot-assisted minimally invasive surgery (da Vinci surgical system) used for the kidney capsule placements required that the hydrogels on the DynaMatrix^®^ in the 3D-ST be rolled with the hydrogel inside so as to fit neatly down the delivery cannula in the patient cart and then be unrolled by the robotic arms on the tissue controlled by the surgeon at the surgeon console. This was accomplished without incident. Two different suturing techniques were tested following the passage of the 3D-ST through the da Vinci delivery port in the patient cart. In the first method, the implant was placed on a cleared section of renal tissue, hydrogel side down, and the DynaMatrix^®^ membrane was stitched to the adjacent renal capsule membrane. In the second method, the renal capsule membrane was gently slit open and the hydrogel slid underneath the membrane, after which the renal membrane was pulled over the 3D-ST implant and stitched back together.

#### Parotid Implantation

The parotid was used for all salivary gland implant studies because of its ready accessibility and high salivary output. In all cases, only the right gland served as the surgical implant site. For studies with irradiated animals, this was also the irradiated side. Once animals were anesthetized, they were placed on their lateral side to expose the head and neck area. The skin was prepped and disinfected with iodine-based disinfectant and 70% (v/v) ethanol. The location of the parotid gland in miniswine of this approximate age was determined previously (Wang et al., 2015); this area was marked on the skin with a vertical incision line. The animal was covered with a sterile surgical drape, and an incision was made in the skin over the parotid using an electrosurgical pencil (Covidien, Medtronic, Minneapolis, MN) with coated electrodes. Deepening the incision to access the parotid gland involved gently moving layer by layer through the epidermis, dermis, and hypodermis, dividing fat layers as needed. The exposed parotid gland was easily recognized by its grapelike appearance, even after irradiation. Coagulation with fine-tipped bipolar forceps controlled bleeding. Dissection in the direction of the facial nerve reduced the risk of injury to the facial nerve. A self-retaining retractor displayed the surgical site. The parotid is a large gland in the miniswine; thus, there was adequate space to create two deep pockets in the exposed parotid, one dorsal and one ventral, into which the two 3D-STs could be placed. The decision of where to place these pockets under the incision line was based upon the surgeon’s assessment of the individual animal’s parotid anatomy and avoiding the facial nerve. The implants were marked using surgical clips placed dorsal, ventral, anterior, and posterior to the 3D-ST. After the two 3D-ST implants were placed, a multilayer closure was performed, essential to precluding wound dehiscence. A ventrally oriented Penrose drain was positioned with a purse string suture through a separate incision prior to closure. A multilayer closure was performed, essential to precluding wound dehiscence. The deep and superficial fascial layers were approximated with an interrupted 3–0 VICRYL^®^ (undyed and braided, Ethicon) suture. Several drops of anesthetic bupivacaine (0.1% v/v, Hospira, Lake Forest, IL) were placed topically prior to a running subcuticular closure using 3–0 monofilament nylon suture (Monosof, Medtronic). The drain was removed at 48 h. Implants were left in the parotid gland for various durations up to 8 weeks.

### Immunosuppression

Subsequent experiments using hS/PC populations encapsulated in hydrogels were carried out in animals beginning an immunosuppression and health regime on the day of surgery. For immunosuppression and health, the animals receive a regimen described in Table 1.

Medications were administered post-surgery in various foodstuffs including peanut butter and dessert treats. Animals were checked several times a day for any signs of infection, malaise, fever, or behavioral changes.

### Tissue Harvest, Handling, and Analysis

At the end of the implant study period, animals were terminated humanely using an AVMA-approved method of euthanasia. The latter consisted of an overdose of intravenous potassium chloride (20–40 mEq/animal) given while under deep anesthesia, followed by puncturing of the heart and continued organ dissection of the carcass. Tissue blocks containing implant-laden renal or salivary tissues (parotid) were excised and shipped overnight on ice from Pittsburgh to Houston. The remainder of the carcass was incinerated.

After receipt, tissue was processed in Houston. Fiduciary markers (surgical clips) were used to locate the sites of the implants. Degrading stitching materials could also distinguish the sites of the implants in tissue. Human cells in the 3D-ST could be located by careful dissection originating at the external suture site and navigating to the approximate implantation depth and location of the surgical clips. Surgical clip orientation and number assisted in differentiating between implanted 3D-STs. A large margin was excised beyond the surgical clips containing any visible non-resorbed DynaMatrix^®^ and hydrogel for processing. Additionally, portions of the right parotid gland adjacent to the implantation site and the left parotid gland were excised for further processing.

Immediate assessment of tissue/cell viability using the Live/Dead assay under confocal and multiphoton microscopy (Nikon A1R/MP, Nikon Instruments) was performed for three regions: 1) with implant, 2) adjacent to implant, and 3) contralateral gland. The same three regions were cryopreserved in O.C.T. compound and sectioned (8 µm) for immunohistochemistry. Biointegration markers were used to assess innervation (beta III tubulin) and vascularization (CD31) and co-localization of secretory α-amylase, and HNA in the implant region was used to locate the viable hS/PCs. Similar analysis of regions adjacent to the implant and the contralateral gland served as controls for irradiated and non-irradiated tissues.

### Saliva Collection and Detection of 3D-ST Products in Saliva

To collect saliva, animals were anesthetized using the isoflurane induction chamber, followed by intubation. An IM injection of pilocarpine (1.5–2 mg/kg) was administered to induce salivary secretion. A cellulose-derived collector was placed over the opening of the right and left parotid ducts in miniswine, and saliva entered the collector by capillary action over a 10-min collection period. Blood could be collected from a peripheral vein while the animal was anesthetized where needed. Collected saliva and plasma was shipped to Houston where GLuc and human α-amylase levels could be assessed as a measure of implant function.

## Results

### Biocompatibility of Cell-Free Hydrogels in the Miniswine Parotid

**FIGURE 1:**
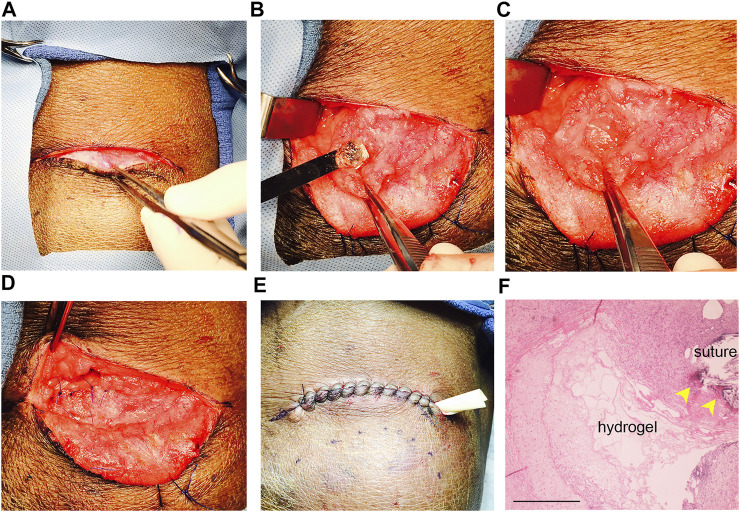
Hydrogel biocompatibility study in the intact, non-irradiated miniswine model. **(A)** Incision site; **(B)** placement of acellular hydrogel (without the SIS membrane) in the untreated parotid bed in a surgically created pocket using a sterile spatula; **(C)** exposed hydrogel in the pocket before closure; **(D)** sutured pocket with enclosed hydrogel implant; **(E)** sutured site of implantation after tissue closure with a ventral drain inserted; **(F)** H & E stained section of tissue removed after 1 week of implant. Note that hydrogel remnants are still detectable near suture sites (yellow arrows). Less inflammation surrounds the hydrogel than the sutures. Scale bar = 500 µm.

Both HA-SH/PEGDA and peptide-modified hydrogels were well tolerated in intact, non-irradiated animals. As shown in Figure 1, surgical placement of an acellular HA-SH/PEGDA hydrogel in the parotid of an intact miniswine (Figures 1A–D) produced minimal tissue reaction after 1 week. Although a drain was inserted (Figure 1E), there was minimal fluid leakage post-surgery. Hematoxylin and eosin (H & E) staining revealed a typical post-surgical level of inflammatory cells including lymphocytes, some macrophages, and a few neutrophils associated with a tissue injury at a surgical site (pathology report, not shown). No excess inflammation was induced by the implanted HA-SH/PEGDA hydrogel, and it was judged to involve lower reactivity than to the sutures themselves (Figure 1F). All subsequent work was conducted in immunosuppressed animals and cell-laden hydrogels.

### Handling and Performance of Pre-Implantation 3D-ST

**FIGURE 2:**
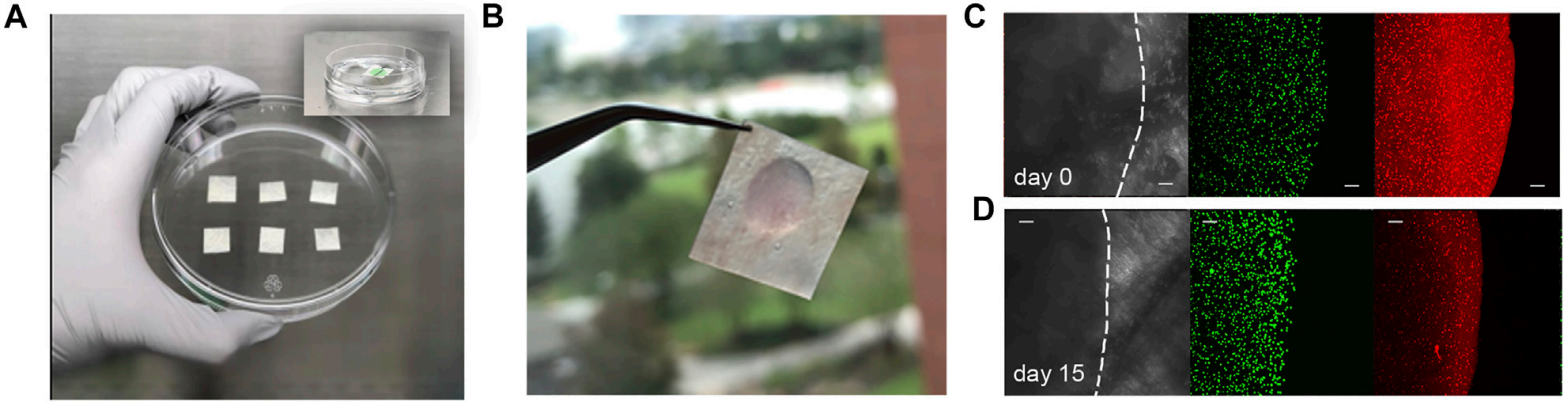
Creating the 3D-ST prototype. **(A)** DynaMatrix^®^ membrane cut into small squares for pilot study; in later versions, these are cut circularly. **(B)** Hydrogels attached stably to the SIS membrane. **(C)** Gelation of hydrogel with encapsulated fluorescent green beads (10 μm) and red beads (200 nm) to visualize hydrogel shape and position on the day of encapsulation and **(D)** after 15 days of mock feeds and handling. The bulk hydrogel and edges remained intact after 15 days. Scale bars = 100 µm.

All cell encapsulations were performed in Houston and then shipped to Pittsburgh. First-generation implants without the DynaMatrix^®^ membrane (seen on the spatula in Figure 1B) proved difficult for the staff and surgeon to handle in the surgical suite. In subsequent studies, hydrogels (3:1/6:1) containing cells were attached to a DynaMatrix^®^ SIS membrane to create the 3D-ST. This membrane is delivered in sheets that can be cut into any shape or size. Figure 2 shows the initial prototype of the 3D-ST using a square SIS membrane (Figures 2A,B) that was modified to a larger circular shape in later experiments for easier handling. This gel-on-SIS is what we refer to as the 3D-ST. This pilot study tested the contact interface between the DynaMatrix^®^ and the hydrogel when cultured over 15 days. The hydrogel in the Figure 2A inset contains fluorescent microspheres (green = 10 μm and red = 200 nm) to visualize the position and the contact perimeter of the hydrogel on the SIS membrane. The 200-nm beads were chosen as they are slightly larger than the porosity of the hydrogels. If degradation of the hydrogel occurs, they will move, and this can be seen. The larger 10-µm beads were chosen to provide fiduciary markers for the bulk hydrogel. Hydrogels attached stably to the SIS membrane and did not detach during handling (Figures 2B,C) or any mock feeding and handling procedures. The hydrogels, approximately 5 mm in thickness, remained intact when evaluated after 15 days (Figure 2D) (dotted line = perimeter of the hydrogel). The smaller red beads show more edge detail of the hydrogel, still intact and coupled to the hydrogel after 15 days. These membrane-stabilized 3D-STs proved to be much easier for the surgical team to handle and could readily be placed in tissue during open surgery or when delivered through the delivery port on the da Vinci surgical system.

**FIGURE 3:**
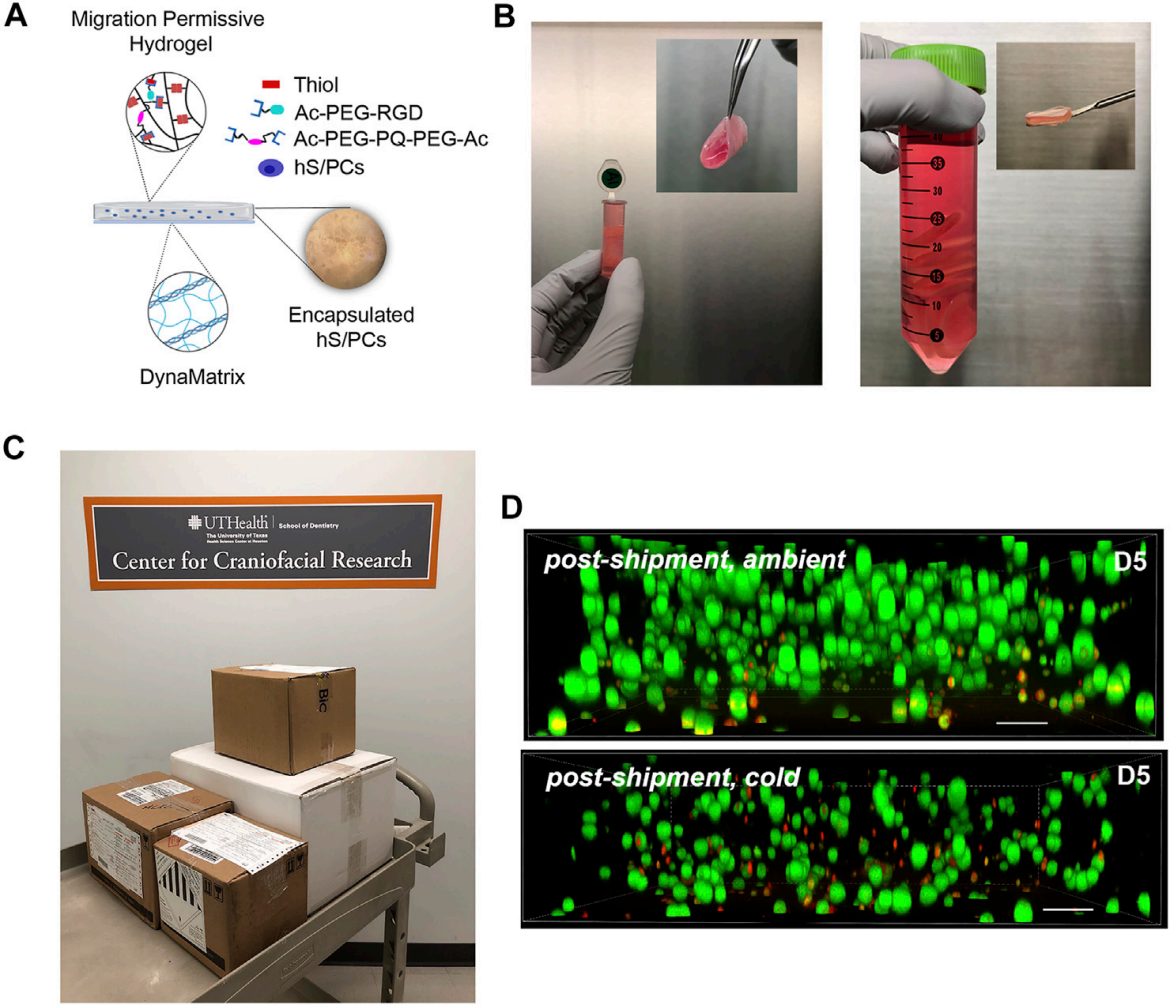
Pre-implantation handling of the 3D-ST. **(A)** Composition of the 3D-ST containing migration-permissive hydrogel on the DynaMatrix^®^ membrane and encapsulated hS/PCs; **(B)** Rolled 3D-ST prepared for shipment for implant on the renal capsule using the da Vinci surgical system (left); stacked 3D-STs prepared for implantation on the parotid in open surgery (right); **(C)** packages prepared for overnight shipment from Houston to Pittsburgh; **(D)** Live/Dead assays performed on encapsulated hS/PCs after shipping priority overnight at ambient (top panel) or cold (bottom panel) temperatures. Green, live; Red, dead. Scale bars = 200 µm.

Shipping protocols for the 3D-STs were determined by an overnight shipment pilot using a commercial carrier to test viability in ambient vs. cold temperatures (Figure 3). Encapsulated and uniformly distributed hS/PCs in migration-permissive hydrogels on DynaMatrix^®^ (Figure 3A) were packaged in fresh culture media after 4 days. For the robot-assisted surgeries on the kidney capsule, the 3D-STs needed to be rolled for delivery *via* the da Vinci delivery port (Figure 3B, left), whereas they were stacked flat for open surgery on the parotid (Figure 3B, right). Figure 3C shows the pre-shipment packaging for all materials being sent from Houston to Pittsburgh. Encapsulated hS/PCs showed higher viability (Live/Dead assay) when shipped at ambient temperatures (Figure 3D, top) than cold (Figure 3D, bottom). Live/Dead assays showed more dead cells (red) and fewer live cells (green) in the cold shipment groups upon return the following day than in the groups shipped at ambient temperatures (Figure 3D). No measurable loss of viable cells was observed, and future 3D-ST shipments were prepped and priority-shipped overnight at ambient temperatures. Quantification of viable cell structures is presented in Supplementary Figure S1.

### Performance of Cell-Laden 3D-ST After Implant in Immunosuppressed Miniswine

Initial experiments with cell-laden hydrogels in immunosuppressed animals were performed using the engrafted kidney capsule, an accepted xenograft site that could assess any signs of rejection prior to testing in the parotid bed. The renal capsule studies were performed using the da Vinci surgical system, and the parotid studies were performed in open surgery. Both methods were well tolerated by the animals, and both surgical methods could be used with the 3D-ST as designed.

#### Kidney Capsule

**FIGURE 4:**
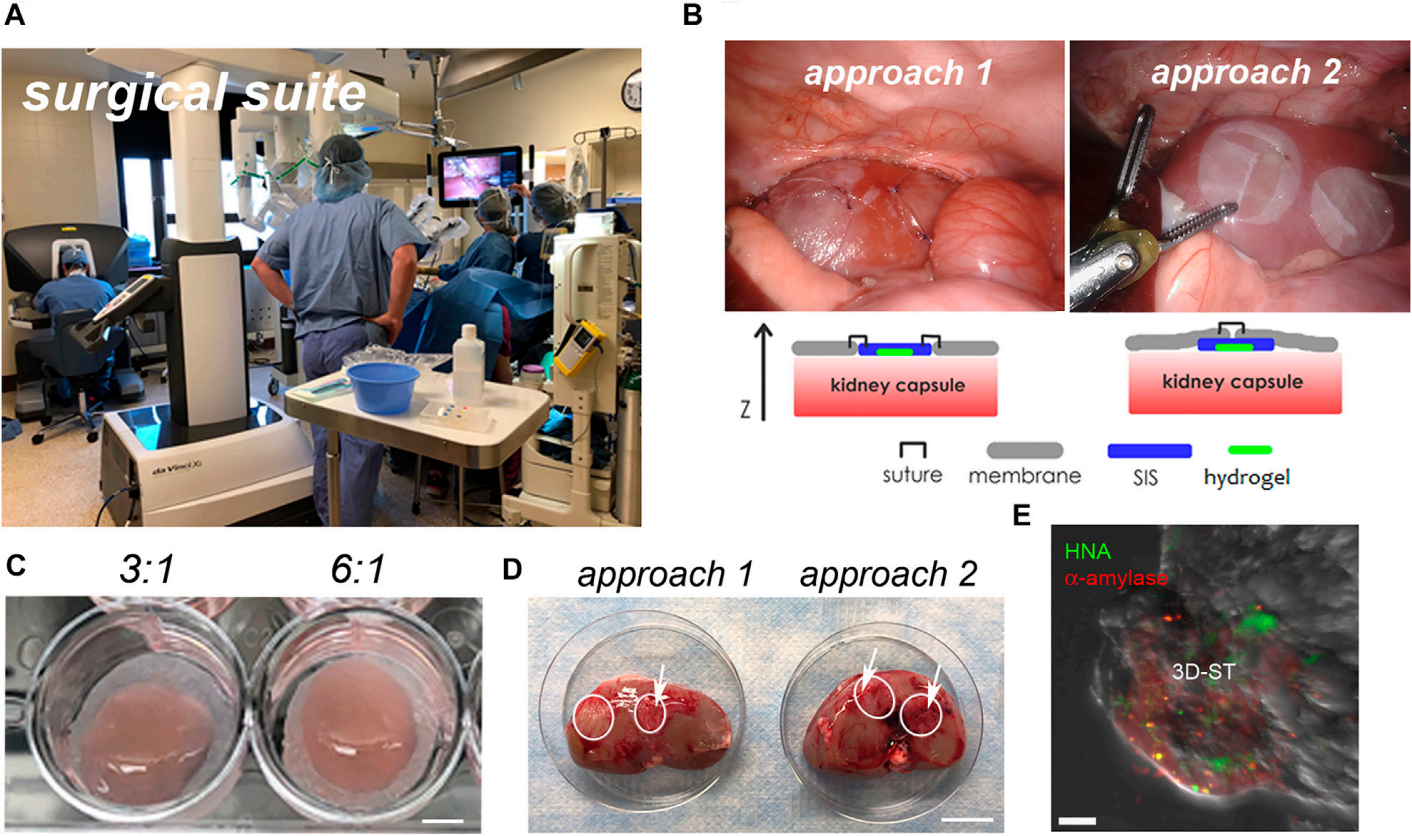
Implantation of 3D-ST onto the renal capsule. **(A)** Surgical suite with the da Vinci surgical system; **(B)** two different approaches to test stitching of sutures; left, the SIS membrane is sutured directly to the renal capsule membrane; right, the 3D-ST is slid into position underneath the membrane of the renal capsule and then the capsule membrane is re-stitched together over the 3D-ST. **(C)** In both cases, the hydrogel containing the encapsulated cells, visible on the DynaMatrix^®^, faces down toward the kidney; 3:1 and 6:1 refer to the ratio of thiol–acrylate (SH-Ac) used in the hydrogel formulation to control stiffness; scale bar = 0.5 cm. **(D)** Resected kidneys after 2 weeks of implant showing 3D-STs (outlined in white); revascularization of implant (white arrows); scale bar = 3 cm. **(E)** Identification of living hS/PCs in 3D-ST on the resected renal capsule by testing for human α-amylase (red) and human nuclear antigen (green); scale bar = 10 µm.

The rolled 3D-ST was applied to the renal capsule *via* the da Vinci delivery port in the surgical suite as seen in Figure 4A. Two methods of suturing the 3D-ST into place were tested (Figure 4B). Suture approach 1, in which the 3D-ST with the hydrogel (Figure 4C, before rolling) face down was stitched directly to the renal capsule membrane, proved to be slower and slightly more difficult than suture approach 2, in which the 3D-ST was slipped in through a slit made in the capsule membrane. Once in, the membrane was re-sutured, and the implant remained in place. We found that sliding the 3D-ST implant under the capsule membrane and quick stitching proved superior to the first method. Neither of the animals showed signs of pain or distress, and the implants and immunosuppression regimen were tolerated well. Figure 4D shows the appearance of the 3D-STs on the resected renal capsule after 2 weeks of implant under immunosuppression. Substantial vascularization of the region surrounding the implants was observed for both configurations. Figure 4E shows the appearance of one of the 3D-STs after retrieval. The hS/PCs remained viable and could be distinguished from the kidney tissue by expression of human nuclear antigen (HNA) and human α-amylase (green and red signals, respectively).

#### Parotid

**FIGURE 5:**
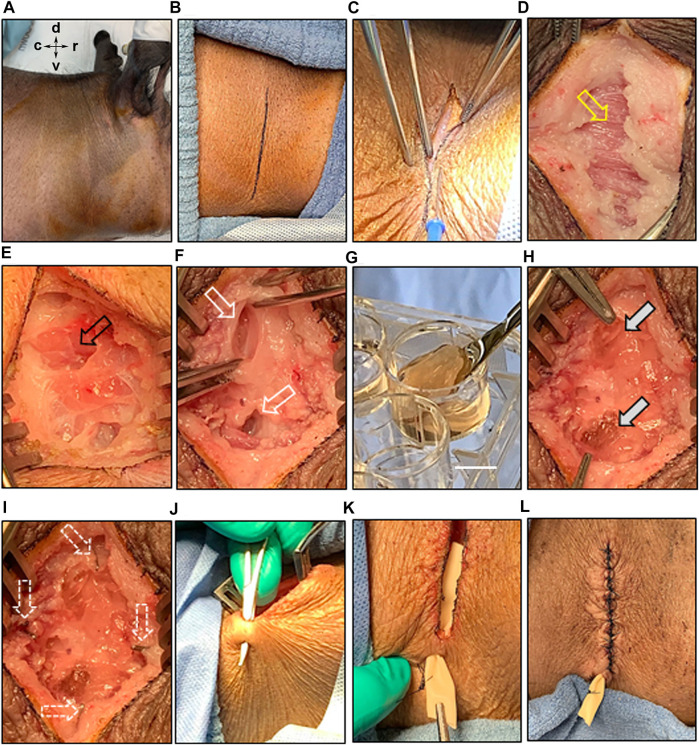
Implantation of 3D-ST into the irradiated parotid bed. **(A)** Prepping of the irradiated surgical site with antiseptic agents; for orientation: dorsal (d), ventral (v), caudal (c), and rostral (r); **(B)** an incision mark was placed on the skin, overlaying the position of the parotid gland; **(B–D)** surgical separation of the various skin layers and the connective layer with platysma fibers (yellow arrow) underneath; **(E)** exposure of the parotid gland (black arrow); **(F)** creation of two pockets into the parotid gland bed; **(G–H)** delivery of the 3D-ST implant under sterile conditions; scale bar = 1 cm, allowing implantation into the glandular pockets (grey arrows); **(I)** multiple ligation surgical clips were placed to orient the location of the two pockets in the gland (white arrows); **(J–K)** ventral to the surgical incision site, a hole was made through the skin to place a Penrose drain, which was sutured to the outside of the skin; **(L)** careful closing of the surgical site by suturing the connective tissue and various skin layers.

Experiments placing 3D-STs in the parotid were conducted in both untreated and irradiated animals, with immunosuppression beginning on the day of surgery in both cases. Figure 5 shows the results of the most recent surgeries we performed. The skin 90 days post-irradiation showed slight discoloration but no signs of blistering or cracking (Figure 5A). For orientation, the dorsal, ventral, caudal, and rostral positions are indicated. The vertical incision line (Figure 5B) drawn on the skin over the parotid allowed the surgeon to readily penetrate the skin (Figure 5C) and continue to dissect down with cauterization as necessary through the tissue layers until the connective tissue layer with platysma fibers was reached (Figure 5D, yellow arrow). Minimal bleeding was observed. When the parotid gland became clearly visible by its texture and appearance (Figure 5E, black arrow), a self-retaining retractor helped keep the surgical site displayed, while two deep pockets, approximately 2.5 cm in size, were developed without removing tissue (Figure 5F, white arrows). These two pockets were created dorsal and ventral to one another in the parotid to allow them to be readily distinguished at harvest. A 3D-ST (Figure 5G) was placed into each pocket (Figure 5H, filled white arrows). Both surgeons who handled the 3D-STs reported that they were reminiscent of natural tissue and were easy to handle in the surgical context. There was sufficient parotid gland to easily accommodate both implants. Ligating surgical clips were placed around the implants to serve as fiduciary markers and for locating the 3D-STs after tissue harvest (Figure 5I, dotted arrows). The closure involved placing the drain and closing the tissue layers very judiciously (Figures 5J–L). The animals well tolerated the surgeries, which took approximately 40 min each, and there were no complications, with stable skin closures post-surgery, when the animals were returned to their pens.

### Biointegration and hS/PC Fate Post-Implant

**FIGURE 6:**
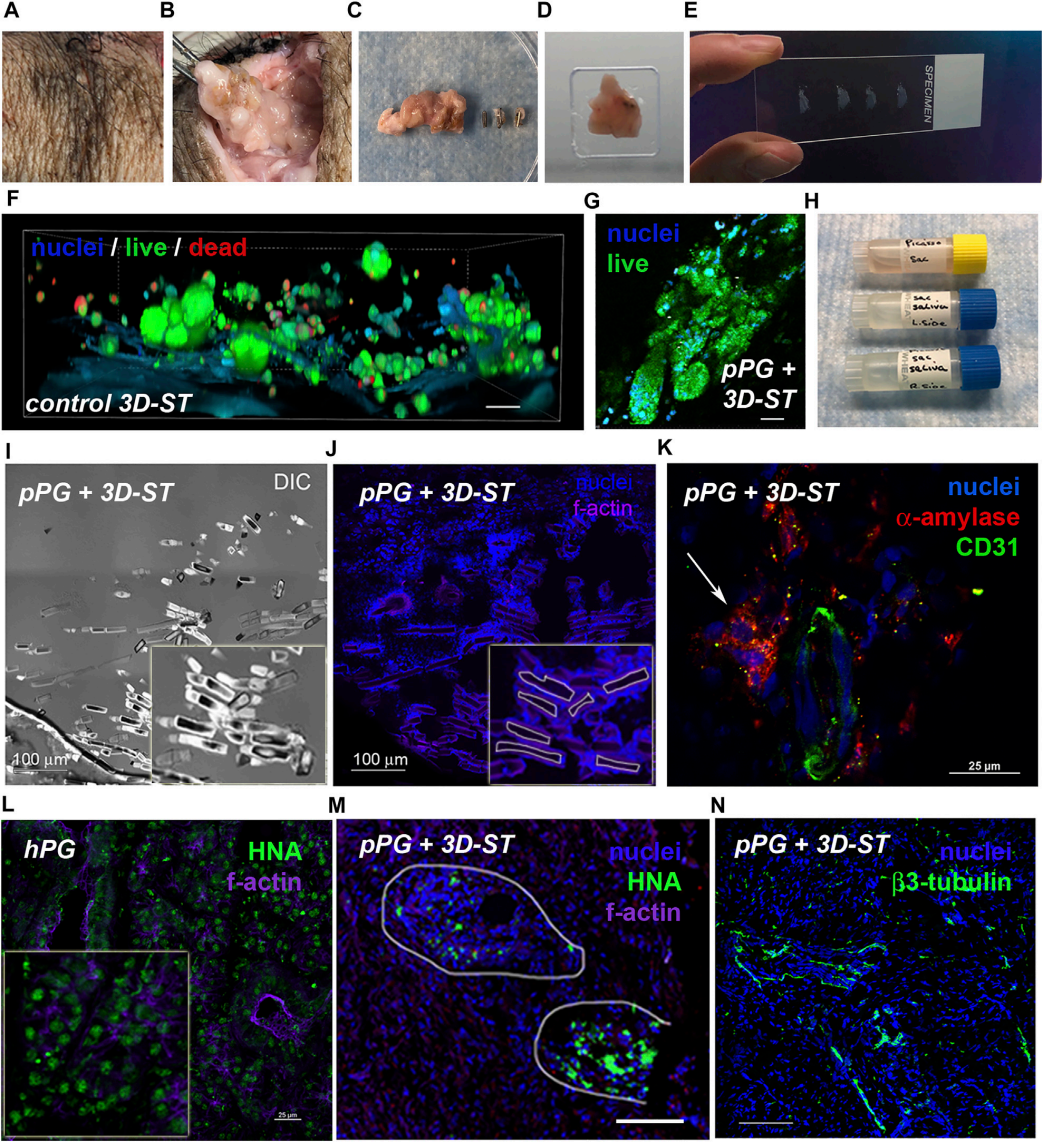
Tissue processing to assess viability and biointegration. **(A)** Tissue is prepared for 3D-ST resection beginning at the closure site; **(B)** 3D-ST implantation site identified using surgical details documented at the time of surgery; **(C)** ligation surgical clips were separated from the resected tissue; **(D)** specimens were embedded in OCT and **(E)** cyrosectioned for immunohistochemistry; **(F)** Live/Dead assays of time-matched *in vitro* controls; scale bar = 25 μm and **(G)** resected tissue 8 weeks post-surgery; scale bar = 25 μm. **(H)** Plasma and saliva collections are used for human α-amylase and GLuc detection; **(I–J)** visualization of resected tissue revealed tissue response (blue nuclei) to surgical sutures; scale bar = 100 μm. **(K)** Vasculature (CD31, green) and human α-amylase (red, white arrow) adjacent to 3D-ST in serial cryosection of human nuclear antigen (HNA^+^) structures ∼256 μm away; scale bar = 25 μm. **(L)** Human nuclear antigen antibody (HNA, green; scale bar = 25 μm) validated using human parotid tissue was **(M)** present in structures in resected implant tissue after 4 weeks; scale bar = 100 μm. **(N)** Nerve fibers in tissue adjacent to implant, ∼448 μm away from HNA^+^ structures, was shown by the presence of beta-III tubulin (green) staining traversing the tissue; scale bar = 100 μm.

No transplant rejection was seen in any animal assessed by visual tissue reaction or redness of the superficial tissues near the site of the implants, and no experiment was terminated early because of implant rejection or infection leading to fever or change of behavior in the animal. After the animals were euthanized, tissues were resected and shipped on ice to Houston by overnight priority without incident. Figure 6 shows key elements of tissue harvest, processing, and analysis. For viability testing, it was critical to immediately begin to process the tissue upon arrival. Both parotids were harvested, the right side containing the 3D-ST in the parotid and the left side, the control parotid gland. The left side tissue was visually normal as seen previously (Wang et al., 2015). By locating the still visible suture site in the treated side (Figure 6A) and following the notes and fiduciary mark documentation from that surgery, it was possible to perform the dissection and locate the 3D-ST and the surrounding clips (Figure 6B). While some degradation of both hydrogel and DynaMatrix^®^ had occurred, even after 8 weeks, it was possible to locate the incompletely degraded SIS membrane and associated hydrogel. A border that extended 0.5 cm radially beyond the ligation clips was followed to resect the tissue for analysis (adjacent tissue area). After the tissue block was isolated and removed (Figure 6C), the clips were separated from the tissue before a portion was embedded in O.C.T., frozen, and sectioned for immunohistochemistry (Figures 6D,E). Live/Dead assays were performed on a separate section of the 3D-ST removed from the animals. A parallel Live/Dead assay was performed on time-matched 3D-STs that remained in culture *ex vivo* for the duration of the implant study (Figure 6D). As shown in Figures 6F,G and Supplementary Figure S2, viability was high in both conditions. Cell assemblies in both implanted and continuously cultured 3D-STs grew larger than those on the implant day. As part of the study protocol, both collected plasma and saliva (from right and left ducts) were shipped from Pittsburgh to Houston for analysis (Figure 6H). Presence of GLuc and/or human α-amylase in saliva can serve as an index of functional implants producing salivary components that reach the oral cavity. Presence in plasma suggests implant viability but a failure to achieve directional flow through the ducts to the mouth. Figures 6I,J shows a DIC image and a fluorescence image (nuclei = blue, filamentous actin = purple), respectively, of suture material after 4 weeks of implantation. As readily seen, degraded sutures were surrounded by cells (blue nuclei in Figure 6J), forming a “wall” separating suture and tissue, an expected foreign body response.

To assess biointegration and 3D-ST cell fate, frozen tissue blocks were cryo-sectioned, fixed, and stained for human origin and biointegration markers. Figure 6K shows staining of a tissue region containing 3D-ST and porcine parotid (pPG). Figure 6L shows a section of the human parotid gland (hPG) from a tissue donor control that stained uniformly for HNA in both acinar and ductal regions (green dots). Tissue in and adjacent to 3D-STs revealed vascularized regions (CD31, green) and human α-amylase (red) in close proximity, ∼256 μm away from the human nuclear antigen positive (NHA^+^) serial section (Figure 6M) indicative of the initial biointegration of a vascular network within the implant. In the pPG containing the 3D-ST (Figure 6M), HNA staining (green) was seen after 4 weeks of implant. Cytoskeletal structures that were counterstained for f-actin (purple) showed that the human cells were nonuniform in distribution and corresponded to regions of residual 3D-ST. Right porcine parotid gland resections containing 3D-ST implants showed regions of beta-III tubulin (green) (Figure 6N) ∼448 μm away from the HNA^+^ serial section, indicative of neoinnervation. Thus, in all experiments, viability of implanted hS/PCs was high, and there were clear signs of both vascular and nervous system integration in the parotid 3D-ST implants.

### Host Animals and Immunosuppression

All animals tolerated immunosuppression for a maximum of 8 weeks of these studies, although a decline in appetite was observed in the last 2 weeks in some animals on immunosuppression for 8 weeks. No major differences were observed in the animal’s health regardless of the irradiation status. Some animals became “wise” to the concealment of their medications in food, so a variety of treats were used and rotated. Some staff noted that as time went by, some of the animals required greater enticements to take the medication, which was especially problematic on weekends when staffing was more restricted. No infections occurred in any animals.

## Discussion

### The Human-in-Miniswine Model Is Suitable for Implant Testing

**FIGURE 7:**
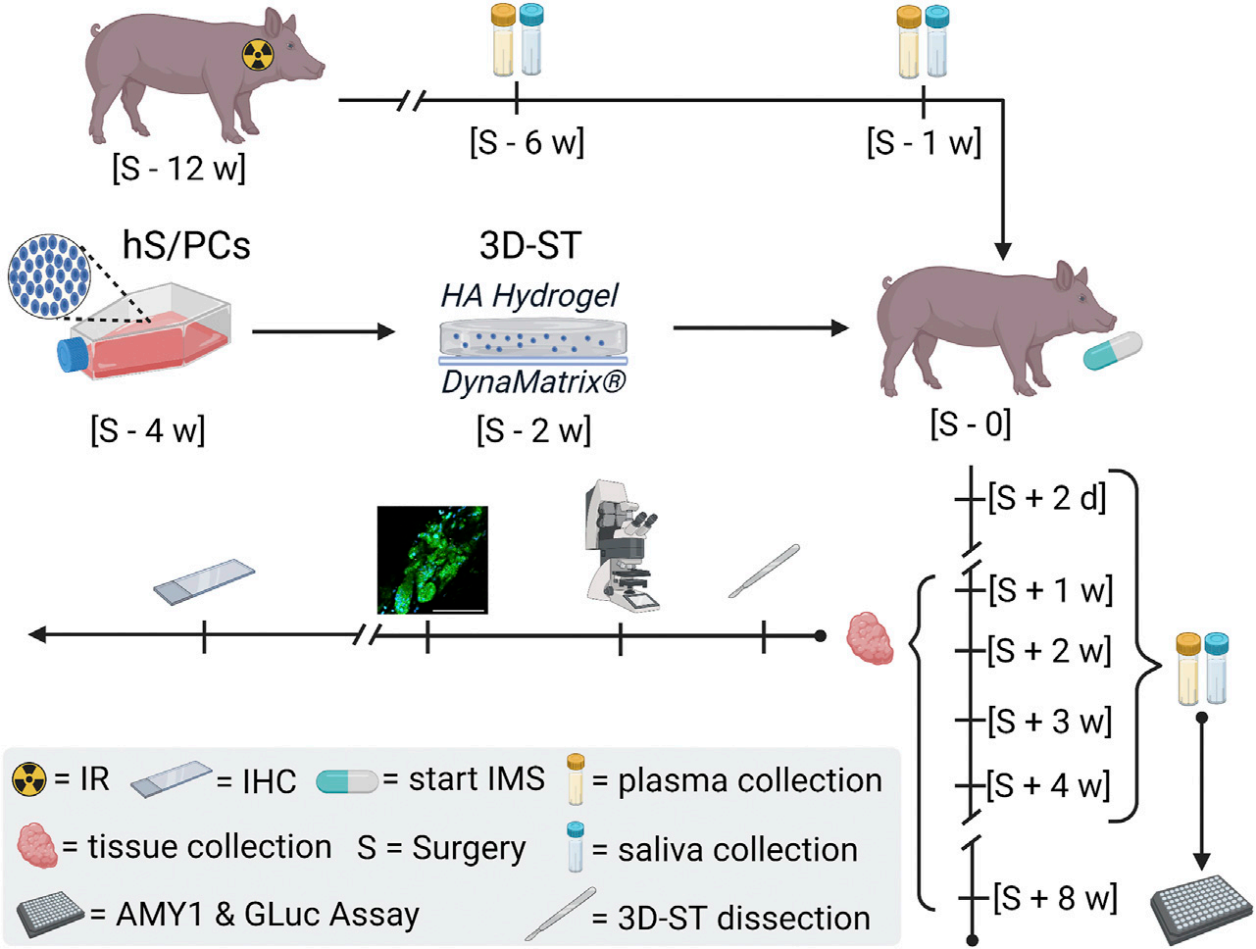
General schematic of the miniswine implant model. Irradiation (IR) of the minipig is scheduled 90 days ∼ [S—12 w] before the day of surgery [S—0] to induce hyposalivation. Saliva and plasma samples are collected post-IR [S—6 w] and [S—1] to assess IR-induced hyposalivation and to establish miniswine-specific baselines. The encapsulation of hS/PCs in implantable 3D-STs is timed for 2 weeks prior to surgery [S—2 w] to ensure cell growth and stability prior to shipment. The 3D-STs are shipped to Pittsburgh for implantation into the irradiated minipigs. Immunosuppression (IMS) regimens started on the day of surgery [S—0] as well as continuous care and oversight provided by the Allegheny General Hospital veterinary team. Saliva and plasma samples are also collected 2 days post-surgery [S + 2 days] and weekly [S + *n* w] for downstream human AMY1 and GLuc analysis. Tissue is harvested at terminal timepoints and shipped to Houston on wet ice for 3D-ST dissection and for control-tissue resections for L/D and IHC analysis.

The work presented here and shown in the schematic version in Figure 7 validates the use of the miniswine model for testing both acellular and cellular implant biomaterials and prototypes designed for relief of xerostomia. While not identical, the salivary system in swine is similar anatomically and functionally to that in humans (Li et al., 2004; Li et al., 2005; Xu et al., 2010). Swine have four paired salivary glands, the parotid, submandibular, submaxillary, and sublingual glands, whereas humans have three paired salivary glands, the parotid, submandibular, and sublingual glands. The swine parotid gland, as in humans, is the largest and most easily visible of all of the salivary glands. As in humans, it lies ventral to the ear and is posterior to the masseter muscles and peripheral facial nerve branches that similarly cross this muscle. Its duct, similar to that in humans, enters the oral cavity buccal mucosa adjacent to the upper molars. The swine submaxillary gland is a bean-shaped gland and lies under the larger parotid gland. The swine submandibular gland, like its human counterpart, lies near the inferior mandibular border, inferior to the parotid salivary gland. It is smaller and almost grape-like in shape, with projections of tissue. Both humans and swine possess sublingual salivary glands, which add to the mucous content of saliva. These glands are similarly situated basal to the tongue, but the ductal structures reaching the oral cavity are somewhat different. Salivary glands in swine produce 15 L of saliva per day, whereas the human salivary gland produces 1 L per day. Swine saliva, like that of humans, contains the following: 1) water for moistening food, 2) mucus (mucin) for lubricating food and binding it into a bolus, 3) salivary amylase to start the breakdown of starch, 4) bicarbonate to buffer acidic food in the mouth, and 5) antibacterial agents to kill oral bacteria, mucin, and lingual lipase. Similar to humans, swine parotid salivary gland innervation includes sympathetic neurotransmitters and the dominant parasympathetic system in which nerves travel from the brainstem by the glossopharyngeal (CN IX) nerve and then *via* branches of the trigeminal nerve (CN V) (Melon, 1975; Wojtkiewicz et al., 2011). Histologically, the swine salivary glands contain acinar cells, myoepithelial cells, and ductal cells, as do human salivary glands.

Miniswine tolerated irradiation well in the doses used in these studies. The time window approximately 90 days post-irradiation proved to be an excellent one for implant testing. This time window is a good compromise between housing costs and humanitarian treatment of the subjects as salivation has slowed, but not ceased, and the animals remain healthy and interested in food as they enter the surgical implant protocol (Wang et al., 2015). We consider that the animals that we implanted had not yet developed the extensive fibrosis often seen in xerostomic patients having undergone head and neck radiation years prior (Pandya et al., 2014). The reported presence of poorly proliferating acinar cell clusters remaining in these patients (Luitje et al., 2021) indicates that this fibrosis may inhibit acinar cell repopulation, a finding that could influence the fate of newly implanted cells as well.

During this work, we tested both acellular and cell-containing biomaterials. Intact miniswine without immunosuppression were found to be excellent models for materials testing. In the past, we performed materials testing on the backs of untreated miniswine, a very different tissue bed than the parotid (data not shown). Here, materials testing was moved to the parotid tissue bed. In untreated animals in which saliva flows freely, it was noted that the tissue pocket created during surgery filled with interstitial fluid and saliva. Because saliva has been reported to contain hyaluronidase (Tan and Bowness, 1968), we were concerned that the hydrogels would dissolve quickly. This was not found to be the case, and we were able to recover gel remnants for weeks post-implant. In dryer, irradiated animals, excess saliva was not a problem and should not be a problem if this work moves to clinical trials in xerostomic humans.

The immunosuppression protocol we used was derived from that used for patients receiving face transplants (Siemionow et al., 2010; Hivelin et al., 2016) and adapted for the miniswine implant model, a model with growing interest for preclinical studies and regenerative medicine (Stricker-Krongrad et al., 2017; Enosawa and Kobayashi, 2019). The protocol was effective and allowed both implant design and biointegration to be assessed. In considering future clinical trials in humans using 3D-ST prototypes, there are several very different xerostomic patient populations who could benefit from hS/PC therapies. The first is the population of patients who are newly diagnosed with a head and neck cancer requiring both surgery and irradiation, and from whom normal salivary tissue could be obtained at the time of surgery, expanded, and used for salivary restoration after the end of cancer treatment. For this group, no immunosuppression should be required if they receive a 3D-ST derived from their own cells as autografts. This group, however, only represents a subset of the tens of thousands of cancer survivors living with radiation-induced hyposalivation disorders. For these survivors, allograft cell therapies would require immunosuppression. While current research is attempting to tolerize patients such as those receiving face transplants (which contain viable salivary tissue) such that they can be weaned from permanent immunosuppression (Hivelin et al., 2016), in the immediate term, immunosuppression is still needed. The results of the miniswine model presented here suggest that 3D-ST allografts combined with immunosuppression could be a viable treatment, at least for those whose hyposalivation is severe enough that they would consider implant and immunosuppression.

### Design Considerations for the Surgical Suite

Our results using miniswine demonstrated that the 3D-ST as designed is a surgeon-friendly material that can be used in open surgery or with robot-assisted surgery using the da Vinci surgical system. The SIS membrane provided a solid support that could be rolled temporarily and easily unfolded on the tissue after delivery through the delivery port. When a tissue site lies on an organ with a capsule, as is the case with the kidney, the 3D-ST can be inserted under the capsule and held in place by the re-stitched capsule membrane. In tissues such as the parotid gland, the 3D-ST can be inserted into surgically created tissue pockets in open surgery. Sterile preparation and handling of the 3D-ST, including during shipping, prior to introduction to the surgical suite resulted in no infections after implantation. For these reasons, we continue to pursue further work to establish functional tissue replacement in the miniswine model prior to moving from pre-clinical to first-in-human trials. All of the insights were obtained using the miniswine in intact and immunosuppressed states and taking advantage of its organ and anatomical similarity to humans.

### The Renal Capsule and Parotid Gland Tolerate Human Salivary Cells in Immunosuppressed Miniswine

The renal capsule is a nearly transparent membranous, viscoelastic sheath (Farshad et al., 1999) composed of strong elastic fibers that are composed of collagens and other matrix proteins including elastin. The parotid lacks this structure but can become extremely tough and fibrous post-radiation (Radfar and Sirois, 2003). In this work, several different surgical techniques were assessed using the second-generation 3D-salivary tissue (3D-ST) for ease and stability both on the kidney capsule and in the capsule-less parotid gland. The irradiated parotid in miniswine 90 days post-irradiation, using this protocol as expected, was found to be less vascular and slightly “tougher” than the unirradiated gland but was not difficult to implant with the 3D-ST after pocket creation. The ready placement and immediate multilayer tissue closure was well tolerated with minimal damage to surrounding tissue, portending well for further development of the 3D-ST in this model. Viability studies showed that human cells remained viable, producing α-amylase, for at least 8 weeks post-implant and under immunosuppression. Initiation of biointegration was evident using biomarkers for neovascular and neoinnervation, without any obvious signs of rejection or host immune response. Thus, immunosuppression did not appear to inhibit host and implant integration in the miniswine model. For this reason, we believe that use of the immunosuppressed miniswine model is a major step toward human-in-miniswine testing of a variety of cell-based implant types.

### Post-Implant Saliva and Tissue Processing

We encountered no difficulties in collection of saliva or plasma from minipigs under sedation, although we point out that the highly trained staff in Pittsburgh is critical to the project’s success. Tissue harvest, locating post-implant 3D-STs, and tissue processing were made simpler by the size of the parotid in miniswine. In previous work, we (Pradhan-Bhatt et al., 2014) and others (Yoo et al., 2014; Mitroulia et al., 2019) used rats and mice, both irradiated and non-irradiated, for testing cell-based regeneration therapies. While useful, the relatively small size of the rat parotid makes all steps more difficult and requires higher animal numbers to assess needed parameters of success. Additionally, the salivary glands are quite different in rodents and humans, with somewhat different locations and proportionate sizes (Yoo et al., 2014); hence, the work is less readily translated to the human surgeon.

## Conclusion

Major strengths of the miniswine model are the size of the organs and glands, similar anatomy, similar tissue response to irradiation, sufficient material for analysis of performance, and tolerance of immunosuppression. The weaknesses of the model are the need for specialized facilities and operating theaters equipped with needed instrumentation, requirement for a skilled staff familiar with handling miniswine, and relatively high cost compared to rodent models. Greater acceptance of this model by the research community could lead to expanded availability of appropriate facilities and a higher use that would reduce costs of these pre-clinical studies. A comparison of our miniswine model to conventional rodent models is provided in Table 2. Of course, the use of a more sentient animal such as the miniswine must be considered in the context of the prevailing ethical norms of the research site where the work would be conducted. We conclude that the immunosuppressed miniswine is a high-value emerging model for testing human cell-based implants prior to first-in-human trials.

**TABLE 2:** Comparison of emerging miniswine and classical rodent model for testing salivary implants.

Feature	Immunosuppressed miniswine	Immunocompromised rodent^a^
Active immunosuppression	Yes	No
Anatomy similar to human^b^	Yes	No
Physiology similar to human	Yes	Somewhat
Scaled to human	Yes	No
Radiation tolerant	Yes	Yes
Compatible with surgical robot	Yes	No
Tolerate implanted human cells	Yes	Yes
Commercially available	Yes^c^	Yes
Require specialized housing	Yes	Yes
Relative cost	High	Medium
Regulatory complexity	High	Medium

a Immunocompromised rodent models in common use include nude rat, nude mouse, athymic mouse, SCID mouse, and NCG mouse (triple immunodeficient).

b For a comprehensive comparison, see (Bode et al., 2010).

c Provided as intact animals that can be used without immunosuppression for cell-free complementary biomaterials testing.

## Data Availability

The original contributions presented in the study are included in the article/Supplementary Material; further inquiries can be directed to the corresponding author.
