# Supplementary material for: Immunosuppressed Miniswine as a Model for Testing Cell Therapy Success: Experience With Implants of Human Salivary Stem/Progenitor Cell Constructs
Source: Front Mol Biosci. 2021 Sep 30;8:711602. doi: 10.3389/fmolb.2021.711602 (PMC8516353; doi:10.3389/fmolb.2021.711602)

**Supplemental Figure 1**: Quantification of live structures, post-shipment at day 5, show ambient temperatures yielded higher viability of hS/PC structures compared to cold shipment conditions. (** p = 0.0029, SD, n = 9, 10)


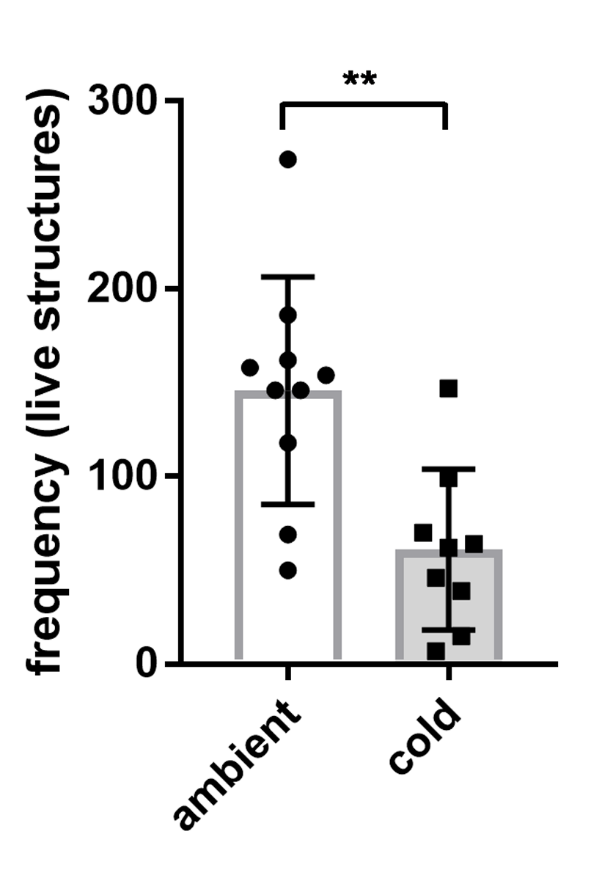


**Supplemental Figure 2:** Surface rendering of Live/Dead image from resected 3D-ST 8 weeks post-implantation. Scale bar = 50 µm.


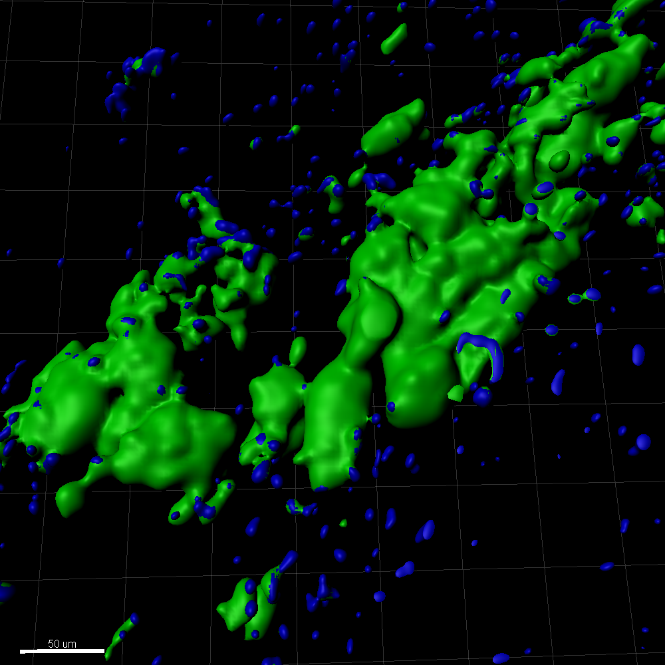

Supplement: Supplementary file 2 [file DataSheet1.DOCX]
